# Hepatocyte generation in liver homeostasis, repair, and regeneration

**DOI:** 10.1186/s13619-021-00101-8

**Published:** 2022-01-06

**Authors:** Wenjuan Pu, Bin Zhou

**Affiliations:** 1 State Key Laboratory of Cell Biology, Shanghai Institute of Biochemistry and Cell Biology, Center for Excellence in Molecular Cell Science, Chinese Academy of Sciences, University of Chinese Academy of Sciences, Shanghai, China; 2 School of Life Science, Hangzhou Institute for Advanced Study, University of Chinese Academy of Sciences, Hangzhou, China; 3 School of Life Science and Technology, ShanghaiTech University, Shanghai, China

**Keywords:** Origin, Hepatocytes, Proliferation, Liver progenitor cells, Liver zonation

## Abstract

The liver has remarkable capability to regenerate, employing mechanism to ensure the stable liver-to-bodyweight ratio for body homeostasis. The source of this regenerative capacity has received great attention over the past decade yet still remained controversial currently. Deciphering the sources for hepatocytes provides the basis for understanding tissue regeneration and repair, and also illustrates new potential therapeutic targets for treating liver diseases. In this review, we describe recent advances in genetic lineage tracing studies over liver stem cells, hepatocyte proliferation, and cell lineage conversions or cellular reprogramming. This review will also evaluate the technical strengths and limitations of methods used for studies on hepatocyte generation and cell fate plasticity in liver homeostasis, repair and regeneration.

## Background

The liver is our body’s largest solid organ, and its multifaceted functions are essential for life. Key liver functions include bile production for fat metabolism, blood filtering, execution of immunological functions, supporting blood clots, absorption and metabolism of bilirubin, storing vitamins and minerals, and synthesis of many hormones. The basic structure is a hexagonal liver lobule, by which central veins are positioned in the center of a hepatic lobule, and portal triads (portal vein, bile duct, hepatic artery) are on the border of the liver lobule. Within the lobule, the primary cell types are parenchymal cells (hepatocytes and bile duct cells), and non-parenchymal cells (endothelial cells, Kupffer cells, fibroblasts, and stellate cells). The microenvironment including nutrients, oxygen, and secreted factors from surrounding non-parenchymal cells varies in different positions of the lobule, resulting in the heterogeneity of hepatocytes (Benhamouche et al. 2006; Burke et al. 2009). Hepatocytes are the main cell type of the liver and are responsible for performing the livers multifaceted functions. With daily wear and tear the liver needs to generate new hepatocytes for maintenance of tissue homeostasis during normal state, or after injuries.

Hepatocytes possess remarkable proliferation capabilities, which are responsible for new hepatocyte generation during homeostasis and regeneration. Nevertheless, when the hepatocytes proliferation is significantly inhibited, bile duct epithelial cells (or cholangiocytes) can contribute to liver regeneration by converting to hepatocytes (Gadd et al. 2020). Due to the heterogeneity of hepatocytes, it remains an intriguing question whether there is a special subpopulation of hepatocytes with higher proliferative capacity during liver homeostasis, repair, and regeneration. Recent studies using genetic lineage tracing reported several distinct yet somehow contradicting models, such as pericentral Axin2^+^ or Lgr5^+^ hepatocytes (Huch et al. 2013; Wang et al. 2015), periportal hepatocytes expressing Sox9 or Mfsd2a (Font-Burgada et al. 2015; Pu et al. 2016), distributed Tert^+^ hepatocytes (Lin et al. 2018), or broadly distributed hepatocytes with predominant proliferation of midlobular hepatocytes (Chen et al. 2020), and the highly proliferative hepatocytes in the midlobular region (He et al. 2021; Wei et al. 2021). In this review, we discuss these different models in detail with considerations for the methods and approaches used in these studies. Molecular mechanisms that regulate hepatocyte proliferation will be examined for a better understanding of the biological processes involved in liver repair and regeneration.

## The epithelial source for new hepatocytes

Hepatocytes and bile duct epithelial cells (or cholangiocytes) are the main components of the epithelium in the liver. In the embryonic liver, hepatoblasts, considered as liver stem cells, are bi-potential and give rise to hepatocytes and bile duct epithelial cells (Zong et al. 2009). However, the existence of facultative liver stem cells (or liver progenitor cells) in adult mouse liver has been heavily debated. In zebrafish, bile duct epithelial cells transdifferentiate into hepatocytes and repopulate the liver after extreme hepatocytes loss (Choi et al. 2014; He et al. 2014; Ko et al. 2019). In the rat, liver progenitor cells were also observed in a 2-acetylaminofluorene (2-AAF)/PHx model, in which hepatocytes proliferation is significantly impaired (Paku et al. 2001). However, this does not translate to mice as mouse liver lacks the *N-sulfotransferase* that activates 2-AAF (Stanger 2015). With the advent of genetic lineage tracing technology in mice, many efforts were made to identify liver stem cells in the past decade. Many studies suggested that liver stem cells appeared around the portal vein and originate from bile duct epithelial cells. Lineage tracing study using *Foxl1-Cre* proposed that Foxl1^+^ cells were liver progenitor cells induced by injury and could give rise to hepatocytes and bile duct epithelial cells (Sackett et al. 2009). Another group generated a mouse line that expressed CreER under the control of the osteopontin (OPN) promoter and demonstrated that OPN-expressing cells are bile duct epithelial cells. Lineage tracing of OPN^+^ cells showed that ductular-derived liver progenitor cells contribute to hepatocytes only in CDE diet-induced injury, but not in other liver injury (Espanol-Suner et al. 2012). An additional study reported that HNF1b is specifically expressed in bile duct epithelial cells in healthy and diseased liver. Lineage tracing of HNF1b^+^ cells using *HNF1b-CreER* demonstrated that liver progenitor cells originating from bile duct epithelial cells could contribute to hepatocytes in chronic injury with LPC expansion, but not in healthy liver or acute liver injury (Rodrigo-Torres et al. 2014). While these studies suggest the existence of liver stem cells, caution should be taken when interpreting the lineage tracing data, and the potential ectopic activity of Cre in hepatocytes should be excluded.

**Fig. 1:**
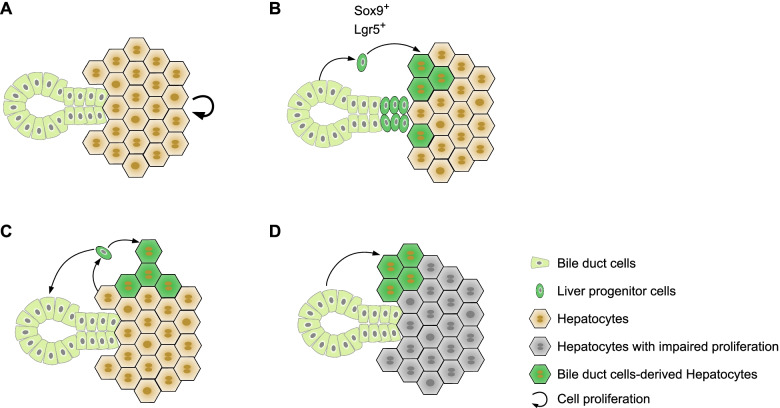
The epithelial source for new hepatocytes. **A** Hepatocytes pool is mainly maintained through self-renewing of preexisting hepatocytes. **B** Liver progenitor cells originated from bile duct cells contributed to hepatocytes. **C** Hepatocytes-derived liver progenitor-like cells converted to bile duct cells and hepatocytes. **D** Bile duct cells contribute to hepatocytes when naïve hepatocytes can’t entry cell cycle

Lgr5^+^ cells act as facultative stem cells in the intestine, colon, stomach, and hair follicles (Barker 2014; Barker et al. 2007). However, the existence of Lgr5^+^ liver stem cells is controversial. Huch et al. proposed that Lgr5^+^ cells induced by liver injury acted as liver stem cells with bi-lineage potential (Huch et al. 2013) (Fig. 1B). Lgr5 was not expressed in normal liver but in a small group of non-hepatocytes close to the bile duct in the injured liver. By performing lineage tracing of Lgr5^+^ cells with *Lgr5-EGFP-IRES-CreER* mice in injured liver, Lgr5^+^ cells were detected in both hepatocytes and cholangiocytes. Single Lgr5^+^ cells isolated from the liver could form organoids that contained both hepatocyte-like cells and cholangiocytes in vitro, indicating bi-lineage potential of Lgr5^+^ cells. After transplantation to *Fah*^*−/−*^ mice, these Lgr5^+^ cells-derived organoids could repopulate the injured liver. Recently, Ang et al. generated *Lgr5-rtTA-IRES-GFP* mice and found these Lgr5^+^ cells were restricted to a subset of hepatocytes located in the pericentral region (Ang et al. 2019). Fate mapping analysis revealed that Lgr5^+^ cells maintained their own hepatocyte population by self-renewal without giving rise to cholangiocytes or expanding to other regions. In the diethylnitrosamine (DEN)-induced tumor models, these Lgr5^+^ cells can contribute to hepatocellular carcinoma. This latest study challenges the view that Lgr5^+^ cells act as in vivo facultative liver stem cells. It also implies that cell plasticity uncovered by the organoid culture or transplantation of cultured cells may not necessarily reflect the in vivo cell fate under physiological conditions.

Sry (sex determining region Y)-box 9 (Sox9) is a member of the box transcription factor family and plays a critical role in embryonic development, tissue homeostasis, and regeneration of multiple tissues and organs (Akiyama 2008; Antoniou et al. 2009; Chaboissier et al. 2004; Stolt et al. 2003). Recently, Furuyama et al. detected Sox9 in bile duct cells but not in hepatocytes. Afterward, they performed lineage tracing of Sox9^+^ cells using *Sox9-IRES-CreER;Rosa26-LacZ* mice and found that 99.4% of LacZ^+^ cells were detected in bile duct cells 1 day after tamoxifen injection, while LacZ^+^ hepatocytes gradually increased over time e.g. 10 and 30 days. They suggested that Sox9^+^ progenitor cells contribute to hepatocytes, which is accelerated by liver injury (Furuyama et al. 2011) (Fig. 1B). One caveat is that absence of hepatocyte labeling at 1 day post-tamoxifen does not mean non-labeling in the following days, as tamoxifen could endure longer than expected. It is possible that hepatocytes may express Sox9, albeit lower than cholangiocytes. A few years later, Tarlow et al. challenged the view that Sox9^+^ cells act as liver progenitor cells (Tarlow et al. 2014a). Clonal analysis of Sox9^+^ cells in different liver injuries demonstrated that Sox9^+^ cells minimally contributed to hepatocytes in vivo. A possible reason for the observation of Sox9^+^ progenitors contributing to hepatocytes by Furuyama et al. is that Sox9 is expressed in a small group of hepatocytes. The labeled hepatocytes could express Sox9 in situ instead of being converted from Sox9^+^ progenitors (cholangiocytes). Indeed, several groups detected a subpopulation of periportal hepatocytes expressing Sox9 (Font-Burgada et al. 2015; Han et al. 2019; Li et al. 2019b). In addition, examination cell labeling 1-2 days after tamoxifen is not sufficient to determine labeling specificity. Sometimes, the duration of tamoxifen required for activating CreER can be a few weeks in some tissues (Reinert et al. 2012). Therefore, we suggest examination of tissues at day 2-3, and 1 and 2 weeks to determine the labelled cell types using CreER.

In zebrafish, it has been reported that all of hepatocytes derived from the embryonic hepatocytes rather than from bile duct cells during homeostasis (Gao et al. 2018). However, the latest researches showed that bile duct cells could differentiate into hepatocytes in homeostasis in zebrafish (Zhang et al. 2021). While in mice, there is increasing experimental evidence indicating that the hepatocyte pool is primarily maintained through self-renewal of preexisting hepatocytes rather than the conversion from liver stem cells during homeostasis and regeneration in adult liver of mice (Malato et al. 2011; Miyajima et al. 2014; Schaub et al. 2014; Tarlow et al. 2014b; Yanger et al. 2014) (Fig. 1A). Malato et al. specifically labeled almost all hepatocytes using AAV8-Ttr-Cre virus. The labeling efficiency was not diluted over time during homeostasis and after injury, indicating hepatocyte renewal was mediated by self-proliferation without a significant contribution from liver progenitor cells (Malato et al. 2011). Yanger et al. used *Krt19-CreER* mice to label cholangiocytes and found they do not contribute to hepatocytes. Similarly, by labeling almost all hepatocytes using AAV8-TBG-Cre virus, they found no significant dilution, indicating new hepatocytes are mainly derived from preexisting hepatocytes (Yanger et al. 2014). At the same time, Tarlow et al. showed that hepatocytes-derived liver progenitor cells contributed to liver regeneration by re-differentiating into hepatocytes (Tarlow et al. 2014b) (Fig. 1C). These lineage tracing studies support hepatocyte proliferation but not facultative hepatic progenitors for new hepatocyte generation. It should be noted that these pathophysiological models are performed in normal adult mouse liver where hepatocytes have strong proliferative capacity.

When new hepatocytes cannot be derived from hepatocyte proliferation, the liver resorts to other cellular sources for hepatocyte generation. When hepatocytes proliferation was experimentally blocked, bile duct epithelial cells were considered as liver stem cells in liver regeneration (Lu et al. 2015) (Fig. 1D). When hepatocyte proliferation was impaired by overexpression of *p21* gene or deletion of *β1-intergrin* gene in injured liver, cholangiocytes could give rise to de novo hepatocytes to repopulate the liver (Raven et al. 2017). These bile duct cell-derived hepatocytes were similar to hepatocytes but transcriptionally distinct from bile duct cells, and account for about 15% of total hepatocytes (Raven et al. 2017). An independent study reported bile duct epithelial cell differentiated to hepatocytes in CDE diet-induced liver injury when hepatocytes proliferation was inhibited by *β-catenin* deletion (Russell et al. 2019). Furthermore, Russell et al. observed few bile duct cell-derived hepatocytes in the early stage of recovery, yet these hepatocytes grow into large clones at later stages, indicating clonal expansion of the initial bile duct epithelial cells-derived hepatocytes without continuous bile duct cell conversion (Russell et al. 2019). Logically, conversion of bile duct cells to hepatocytes is not continuous. Stimulus from injury is absent at the late stage of recovery and the new hepatocytes that have emerged can function and expand accordingly. Chronic severe human liver diseases are usually accompanied by inflammatory infiltration, fibrosis, and hepatocyte senescence (Marshall et al. 2005; Richardson et al. 2007). However, the general injury models used on mice do not mimic human chronic liver injury. Deng et al. recapitulated human liver disease in mice by administrating TAA for 24 to 52 weeks. In this chronic injury setting, they detected hepatocyte regeneration from bile duct cells (Deng et al. 2018). Subsequently, another group induced severe chronic liver injury by duration of CCl_4_ injection and showed bile duct cells converted to hepatocytes clonally (Manco et al. 2019). Bile duct epithelial cell-derived hepatocytes have superiority in proliferation and DNA repair and were resisted to give rise to preneoplastic nodules (Manco et al. 2019). These studies suggested that conversion of hepatocytes from bile duct cells might occur in human chronic liver disease when hepatocytes have reduced proliferation capacity. Identifying the cellular sources of hepatocytes regeneration in the setting of chronic diseases would provide insights for developing new therapeutic strategies.

## Other non-epithelial cell sources for hepatocytes

It has been reported that bone marrow cells contribute to hepatocytes through cell fusion. Two groups transplanted bone marrow cells into fumarylacetoacetate hydrolase (Fah) deficient mice and found bone marrow cells contributed to hepatocytes significantly through cell fusion and rescued liver function (Vassilopoulos et al. 2003; Wang et al. 2003). These bone marrow cells adopt the phenotype of hepatocytes by possessing the morphology of mature hepatocytes and expressing hepatocytes maker Fah. The bone marrow morphology and cell maker CD45 were lost after transplantation. Furthermore, these experiments suggested that repopulated hepatocytes were produced by fusion of bone marrow cells and hepatocytes rather than transdifferentiation. Independent work demonstrated that bone marrow cells not only fused with hepatocytes but also with cardiomyocytes and neurons after transplantations (Alvarez-Dolado et al. 2003). Alvrarez-Dolado et al. transplanted bone-marrow-derived cells into irradiated mice rather than Fah deficient mice and found that bone-marrow-derived cells could fuse with neurons, cardiomyocytes and hepatocytes to form multinucleated cells after transplantation. But this spontaneously cell fusion is limited. Further studies suggested that macrophages or their highly proliferative progenitors possess the most potential for fusion with hepatocytes and therapeutic ability (Willenbring et al. 2004). Recently, Pu et al. found that endothelial cells also could contribute to hepatocytes. By constructing Hep-EC-DeaLT system that permitted simultaneous labeling of hepatocytes and endothelial cells with two distinct genetic markers, they showed that endothelial cells contribute to hepatocytes via cell fusion (Pu et al. 2020). These studies suggest that other non-epithelial cell lineages contributed to hepatocytes minimally in homeostasis but contributed for liver repair and regeneration significantly in very specific experimental conditions.

It has been reported that fibroblasts could be reprogrammed in vitro to induced hepatocyte-like cells that have multiple hepatocyte-specific features and the ability to repopulate in the injured liver after transplantation (Huang et al. 2011; Sekiya and Suzuki 2011). In addition, another study reported that resident myofibroblasts could be reprogrammed in situ to hepatocytes by defined transcription factors (Rezvani et al. 2016), which attenuates liver fibrosis and generates new functional hepatocytes. This in vivo direct lineage conversion from myofibroblasts to hepatocytes provides new insights into a potential therapeutic approach for liver regeneration. Taken together, these studies suggest that other non-epithelial cell lineages, through various mechanisms, can be programmed or induced to generate new hepatocytes for liver repair and regeneration.

## Hepatocyte proliferation

Hepatocytes within liver lobule are similar in morphology but functionally and molecularly heterogeneous depending on their position along the portal-central axis of the liver lobule, which creates a metabolic zonation (Gebhardt 1992). Based on molecular markers and metabolic functions, the liver lobule can be roughly divided into 3 distinct zones: periportal zone (zone 1), pericentral zone (zone 3), and the midlobular zone (zone 2) between zone 1 and zone 3. The microenvironment including nutrients, oxygen, and secreted factors from surrounding non-parenchymal cells varies in different zones of the lobule, resulting in different functions and responses of hepatocytes to tissue homeostasis and injuries (Benhamouche et al. 2006; Burke et al. 2009).

**Fig. 2:**
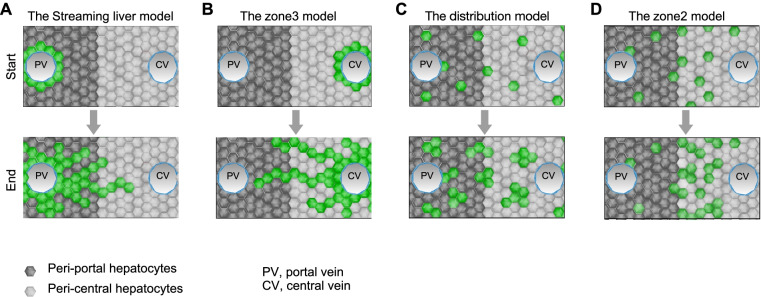
Models for hepatocytes renewal. **A** The streaming liver model proposes that hepatocytes around the portal vein stream to the central vein over time. **B** The zone 3 model supports that a subset of hepatocytes close to the central vein expand during tissue homeostasis. **C** The distribution model suggested that hepatocytes distributed throughout the lobule are equally proliferative without preferential proliferation in restricted zone. **D** The zone 2 model implies that hepatocytes located in the midlobular zone are the main source of new hepatocytes pool

Studies in the early 1980s labeled hepatocytes with tritiated thymidine and revealed that hepatocytes around the portal vein slowly streamed to the central vein during liver homeostasis (Zajicek et al. 1985) (Fig. 2A). Several years later, by genetic labeling hepatocytes using retroviral mediated gene transfer, other groups reported that the hepatocyte renewal occurred throughout the lobule in homeostasis, providing evidence against the streaming of hepatocytes (Bralet et al. 1994, 1995; Zajicek 1995). With the advent of Cre-loxP-mediated genetic lineage tracing, Wang et al. used *Axin2-CreER* line to label pericentral Axin2^+^ hepatocytes 2 days after tamoxifen treatment (Wang et al. 2015). After 1 year of tracing, these initial labeled pericentral hepatocytes expanded significantly along the lobule and contributed to up to 40% of the hepatocytes during homeostasis (Fig. 2B). In some lobules, these labeled hepatocytes spread to the portal vein and expressed makers for periportal hepatocytes, indicating hepatocyte subtype reprogramming. The authors also found that these Axin2^+^ hepatocytes were mostly diploid and the expansion of Axin2^+^ hepatocytes was regulated by Wnt signals produced by central vein endothelial cells (Wang et al. 2015). Recently, Sun et al. used another *Axin2-CreER* line to label Axin2^+^ hepatocytes and found that they didn’t expand significantly over time (Sun et al. 2020). Of note, they found a slight increase in the number of labeled cells between day 1 and day 7, but no increase in the number of labeled cells between day 7 and 10 months. They suggested that the increase between day 1 and day 7 was induced by the persistence of tamoxifen that continuously induced hepatocyte labeling (Sun et al. 2020), which was overlooked by the previous study (Wang et al. 2015). An alternative explanation for the inconsistency in results between the two groups is the discrepancy between the *Axin2-CreER* lines. Wang et al. generated *Axin2-CreER* line by insertion of CreER into the translational start site of the Axin2 gene to disrupt the endogenous Axin2 gene, resulting in haploinsufficiency (Wang et al. 2015). Sun et al. generated *Axin2-CreER* line as transgenes, keeping the endogenous Axin2 gene unbroken. Disrupting Axin2 genes for knock-in strategy might cause activation of Wnt signaling, possibly leading to enhance proliferation of Axin2^+^ pericentral hepatocytes; while the transgene strategy using a fragment of the promoter to drive Cre may not fully recapitulate endogenous regulatory elements. In the future, it would be helpful to generate an *Axin2-2A-CreER* mouse line by inserting CreER cassette after the Axin2 gene, thus using endogenous gene regulation machinery while keeping Axin2 gene expression intact. In addition to Axin2^+^ pericentral hepatocytes, other lineage tracing studies using *Lgr5-CreER* and *GS-CreER* to label pericentral hepatocytes reported that that the hepatocytes in zone 3 were restricted to the pericentral zone without significant expansion to other regions during liver homeostasis. This indicates that there was no superior proliferation capability of pericentral hepatocytes in liver homeostasis (Ang et al. 2019; He et al. 2021; Wei et al. 2021).

It has been reported that diploid hepatocytes have an advantage in proliferation than polyploidy hepatocytes (Wilkinson et al. 2019). Polyploid hepatocytes could reduce the incidence of tumor formation (Zhang et al. 2018). Axin2+ pericentral hepatocytes were mostly diploid (Wang et al. 2015). However, hepatocytes in zone 3 didn’t display any advantage in proliferation than the hepatocytes in other zones. A recent study showed that polyploid hepatocytes could proliferate by reducing their ploidy after transplantation (Matsumoto et al. 2020).

Lineage tracing of periportal hepatocytes (expressing *Mfsd2a*) revealed that Mfsd2a^+^ hepatocytes decreased during liver homeostasis (Pu et al. 2016). Nevertheless, Mfsd2a^+^ hepatocytes could repopulate the entire lobule in chronic injury induced by CCl_4_ and expressed makers for pericentral hepatocytes to re-establish metabolic zonation. The number of *Mfsd2a-CreER* labeled periportal hepatocytes are significantly reduced when injury, such as BDL (bile duct ligation), occurs in periportal region (Pu et al. 2016). Front-Burgada et al. reported a subpopulation of periportal hepatocytes expressing Sox9 (Font-Burgada et al. 2015), previously identified as a marker for cholangiocytes. By taking advantage of dual recombinases (Cre and FLPo), they specifically labeled a subset of hepatocytes that expressed Sox9 but not any cholangiocytes. These Sox9-expressing hybrid hepatocytes constitute ~ 5% of the total hepatocyte population and possessed extraordinary proliferation ability in chronic damage (Font-Burgada et al. 2015). These periportal hybrid hepatocytes expand from portal vein to central vein in chronic damage induced by repetitive administration of CCl_4_. Of note, hybrid hepatocytes do not contribute to hepatocellular carcinoma compared to the remaining hepatocytes indicating their therapeutic potential (Font-Burgada et al. 2015). These papers indicated that different regeneration stimuli (BDL, partial, toxic liver injury) trigger different hepatocyte subpopulations to proliferate. For instance, CCl_4_ is mainly metabolized by enzyme expressed in pericentral hepatocytes, therefore causing the death among pericentral hepatocytes and promoting periportal hepatocytes to proliferate. While BDL-induced cholestasis induced inflammation and cell death in periportal region, this triggers the proliferation of hepatocytes in other zones (Pu et al. 2016).

A distributed model (Fig. 2C) of hepatocyte renewal was first described by Planas-Paz et al., who showed that Lgr4^+^ hepatocytes were located throughout the lobule and contributed to homeostasis without zonal dominance (Planas-Paz et al. 2016). Subsequently, Lin et al. reported that a subpopulation of hepatocytes highly expressing telomerase reverse transcriptase (*Tert*) was scatted throughout the liver lobule. Lineage tracing of Tert^high^ hepatocytes demonstrated that Tert^high^ hepatocytes comprised 3% of the liver area and could expand to about 30% of the liver area after 1 year’s tracing during liver homeostasis. The expansion of Tert^high^ hepatocytes was accelerated in liver injury. Also, ablation of Tert^high^ hepatocytes in DDC-induced injury resulted in more serious liver fibrosis and collagen deposition, indicating that Tert^high^ hepatocytes were essential to liver regeneration (Lin et al. 2018). A recent study from Willenbring’s group based on random lineage tracing showed that in liver homeostasis proliferating hepatocytes can be found in all zones but are enriched in the midlobular zone (Chen et al. 2020). Chen et al. used AAV8-TBG-Cre virus and *Rosa26-Rainbow* mice to sparsely label hepatocytes. After 13 months of tracing in homeostatic liver, 90% of labeled cells remained single cells, 9% of clones contained 2 cells, and 1% of clones consisted of > 2 cells. Clones >2 cells were mostly located in the midlobular zone; pericentral and periportal clones almost exclusively consisted of 1 or 2 cells (Chen et al., 2020).

Recently, two groups, using different methods to examine hepatocytes in all zones, reported that a subpopulation of hepatocytes located in midlobular zone preferentially contributed to new hepatocytes during liver homeostasis and regeneration (He et al. 2021; Wei et al. 2021) (Fig. 2D). Wei et al. systematically constructed 11 different inducible Cre drivers that distinctly labeled different hepatocytes subpopulations across the lobule, and compared the proliferation of different zonal hepatocytes side-by-side. By using *Gls2-CreER* mice that marked hepatocytes around the periportal zone, and *Cyp1a2-CreER* or *Oat-CreER* mice that marked hepatocytes located in zone 2 and zone 3, they observed periportal hepatocytes decreased while zone 2 and zone 3 hepatocytes increased during homeostasis. To further compare the proliferative ability between zone 2 and zone 3, *GS-CreER* mice that labeled hepatocytes adjacent to the central vein were used, and the percentage of *GS-CreER* labeled hepatocytes didn’t increase or decline. Lineage tracing using *Arg1.1-CreER* mice that labeled almost all hepatocytes apart from GS^+^ hepatocytes further confirmed the long life span of GS^+^ hepatocytes. These findings suggest that zone 2 hepatocytes expand in liver homeostasis, while GS^+^ hepatocytes are maintained by self-renewing without expanding to other zones. To further confirm this hypothesis, Wei et al. generated *Hamp2-CreER* mice that mainly marked hepatocytes occupying the midlobular zone. After 1 year of tracing, early labeled cells comprising 7.4% of the liver area expanded to about 27.4% of the liver area, confirming that zone 2 hepatocytes mainly contribute to the source of new hepatocytes during liver homeostasis (Wei et al. 2021). Interestingly, Wei et al. performed lineage tracing using the same *Axin2-CreER* mice used by Wang et al., (Wang et al. 2015) and observed the expansion of Axin2^+^ hepatocytes. Axin2^+^ hepatocytes occupied 1.2% of the area at 1 week after tamoxifen treatment and expanded to 9.3% of the liver area after 6 months of tracing. Detection of hepatocyte proliferation by EdU incorporation revealed that there were comparable EdU-positive hepatocytes between zone 3 and zone 2 in *Axin2-CreER* mice, while EdU-positive hepatocytes were mainly distributed in zone 2 in other CreER mice (Wei et al. 2021). These results suggest haploinsufficiency of the Axin2 gene might change the proliferation ability of Axin2^+^ hepatocytes, leading to the expansion of zone 3 hepatocytes in *Axin2-CreER* mice compared with other *CreER* mice.

Independently, He et al. developed a new genetic method, proliferation tracer (ProTracer), which enables continuous recording of in vivo cell proliferation (He et al. 2021). ProTracer was based on two orthogonal site-specific recombinases (Cre and Dre). Ki67, widely used as a marker for proliferation, is expressed when a cell enters the cell cycle. However, detection of Ki67 expression only provides a snapshot of cell proliferation at one time point. Recording all cell proliferation in a timeframe that spans from weeks to months during tissue homeostasis is more informative. To achieve this, He et al. generated *Ki67-Cre-rox-ER-rox (Ki67-CrexER)* mice in which ER is flanked by two rox sites. By crossing with *R26-DreER* mice, Dre-rox recombination would remove ER after tamoxifen treatment, which converts *Ki67-CrexER* into *Ki67-Cre* genotype to continuously record cell proliferation thereafter in DreER-expressing cells (He et al. 2021). By using ProTracer, He et al. found that hepatocytes in zone 2 were more proliferative than their counterparts in other zones during homeostasis. Additionally, combined with a tissue-specific promoter, ProTracer could provide high spatial resolution of the proliferation of one specific cell lineage. Furthermore, ProTracer could record cell proliferation non-invasively for a long time in a live mouse. In the future, ProTracer could be applied to study cell proliferation in other fields to understand cell generation during tissue homeostasis and after injuries.

Collectively, the above lineage tracing studies using different lineage tracing strategy provide different conclusions about the origin of hepatocytes renewal. A possible interpretation for the inconsistency in results between the groups is the discrepancy in the strategies for constructing CreER mouse lines. Another possible explanation is the limitation of technology used by different groups. These groups only focused on the expansion of a subset of hepatocytes, which lacks direct comparisons of different hepatocytes subpopulations’ expansion side by side.

## Molecular mechanisms of liver regeneration

### Mechanisms of liver zonation

The mechanisms regulating liver zonation and hepatocyte function remain largely unknown. Wnt signaling has been reported as a major regulator of liver zonation (Burke et al. 2009; Planas-Paz et al. 2016; Sun et al. 2021). Under normal conditions, the ß-catenin gene is activated in the pericentral hepatocytes, and Wnt target genes are also located in pericentral zone. Adenomatous polyposis coli (APC) is a negative regulator of Wnt signaling and is highly expressed in the periportal hepatocytes, therefore suppressing Wnt signaling (Benhamouche et al. 2006). Deletion of the ß-catenin gene causes the loss of pericentral gene expression and activation of periportal genes. In contrast, after the deletion of the APC gene, the whole liver lobule acquires the pericentral genes and loses the periportal genes (Benhamouche et al. 2006). Recent reports suggest that endothelial secreted Wnt ligands regulate liver zonation (Ma et al. 2020; Preziosi et al. 2018). While aberrant Wnt signaling disrupts zonation gene expression, deletion of c-Myc gene didn’t change the expression pattern of liver zonation-related genes (Burke et al. 2009). Other groups suggest that the Ha-Ras pathway is involved in activating pericentral genes expression while suppressing periportal gene expression (Hailfinger et al. 2006; Unterberger et al. 2014). Recently, Fitamant et al. found that Yap plays an important role in maintaining liver zonation by suppressing pericentral gene expression (Fitamant et al. 2015). To better understand the molecular regulation of zonation genes and hepatocyte function, genetic targeting of hepatocytes in different zones is required for further analysis of gene function.

### Mechanisms of hepatocytes proliferation

It’s well known that hepatocyte renewal occurs through replication of preexisting hepatocytes (Malato et al. 2011; Miyajima et al. 2014; Schaub et al. 2014; Tarlow et al. 2014b; Yanger et al. 2014), regulated by a number of complex pathways. Here, we briefly introduced some important pathways related to hepatocytes proliferation. Wnt signaling is a well-known pro-proliferation signal and plays an essential role in promoting hepatocytes proliferation during homeostasis and regeneration (Planas-Paz et al. 2016; Russell and Monga 2018; Sun et al. 2021). Central vein endothelial cells secret Wnt ligands such as *Wnt2* and *Wnt9b* for pericentral hepatocytes proliferation in homeostasis (Wang et al. 2015). RSPO could promote hepatocytes proliferation by increasing Wnt signaling through binding to LGR4-6 receptors in liver homeostasis. These studies also reported that ZNRF3/RNF43 balance Wnt signaling to restrict hepatocytes proliferation, while preserving metabolic function. Deletion of ZNRF3 and RNF43 induced uncontrolled Wnt/b-catenin activity, enhancing hepatocytes replication and ultimately promoting liver tumors. Of note, it appears that proliferating hepatocytes down-regulated the expression of metabolic genes (Planas-Paz et al. 2016; Sun et al. 2021). Recently, the IGFBP2-mTOR-CCND1 axis was reported to mediate zone 2 hepatocytes expansion. CCND1 is primarily expressed in zone 2 hepatocytes, and ablation of CCND1 expression significantly inhibits zone 2 hepatocyte proliferation (Wei et al. 2021). Wnt signaling also contributes to liver regeneration after partial hepatectomy (PH) by regulating cyclin-D1 (CCND1) gene expression (Preziosi et al. 2018). Decreasing the expression of the Wnt signaling delayed but did not abolish liver regeneration after PH (Yang et al. 2014). Epidermal growth factor (EGF) and hepatocyte growth factor (HGF) are the key mitogenic signals for hepatocytes replication after PH. Deletion of the EGFR gene in the adult liver resulted in reduced CCND1 expression and delayed regeneration but did not affect liver function (Natarajan et al. 2007). HGF/Met is also essential for liver regeneration. Met-deficient liver exhibited decreased proliferation, but compensatory mechanisms allow for liver regeneration (Borowiak et al. 2004). Combined elimination of EGF and Met signaling completely inhibited liver regeneration, indicating that EGF and HGF signaling cooperate to regulate hepatocytes replication (Paranjpe et al. 2016). In detail, when both of MET and EGFR signaling pathways were eliminated, many essential hepatocyte functions including metabolism and cell replication were disordered, causing that hepatocytes reverted to 35% of their original volume. The combined signaling of EGFR and HGF/MET provides the basic platform on which all the other signaling pathways of hepatocytes depend. Another important pathway controlling hepatocyte proliferation is Hippo signaling. Hippo signaling regulates cell proliferation in multiple tissues through the transcriptional co-activator Yap1. Overexpression of Yap1 in hepatocytes increased liver size by promoting hepatocytes proliferation, and eventually leading to liver cancer. (Camargo et al. 2007; Lu et al. 2010; Misra and Irvine 2018; Yimlamai et al. 2014). Furthermore, Hippo and Wnt pathways may cooperate to precisely regulated cell proliferation (Jiang et al. 2020; Li et al. 2019a; Varelas et al. 2010).

## Conclusions

The liver can adopt different strategies for repair and regeneration according to the extent of the injury. Hepatocyte proliferation is the dominant cellular mechanism for liver regeneration in the majority of injuries. In conditions where hepatocyte proliferation is impaired, bile duct epithelial cells can transform into hepatocytes and contribute to liver regeneration. For hepatocyte proliferation, there are many different models supported by different genetic tracing tools. The latest reports support that zone 2 hepatocytes mainly contribute to the source of new hepatocytes. Nevertheless, there are still many questions on the proliferation potential of zone 2 hepatocytes, e.g., the underlying molecular mechanisms that provide zone 2 hepatocytes with higher proliferation potential, and the crosstalk of hepatocytes with the surrounding environment for regulation of cell proliferation. Currently, it’s unclear which non-parenchymal cells maintain the potential niche for zone 2 hepatocyte proliferation. In addition, the mechanisms controlling bile duct epithelial cell to hepatocyte conversion remains largely unknown. Do bile duct epithelial cells first transform into a liver progenitor cell status and then differentiate into hepatocytes? What are the surrounding cell types that provide critical microenvironmental cues to promote cell conversion? Further studies focusing on these unsolved, yet important and intriguing questions would provide new insights for the treatment of liver diseases, and would also uncover new therapeutic targets for the treatment of liver diseases.

## Data Availability

Not applicable.
