# Correction to: Hepatocyte generation in liver homeostasis, repair, and regeneration

**DOI:** 10.1186/s13619-022-00115-w

**Published:** 2022-03-23

**Authors:** Wenjuan Pu, Bin Zhou

**Affiliations:** 1 State Key Laboratory of Cell Biology, Shanghai Institute of Biochemistry and Cell Biology, Center for Excellence in Molecular Cell Science, Chinese Academy of Sciences, University of Chinese Academy of Sciences, Shanghai, China; 2 School of Life Science, Hangzhou Institute for Advanced Study, University of Chinese Academy of Sciences, Hangzhou, China; 3 School of Life Science and Technology, ShanghaiTech University, Shanghai, China

**Correction to: Cell Regen 11, 2 (2022)**

**https://doi.org/10.1186/s13619-021-00101-8**

Following publication of the original article (Pu and Zhou 2022), some errors were identified in the Background and Hepatocyte proliferation sections.

The updated three sentences have been highlighted in **Bold typeface**.

**Background**

Recent studies using genetic lineage tracing reported several distinct yet somehow contradicting models, such as pericentral Axin2+ or Lgr5+ hepatocytes (Huch et al. 2013; Wang et al. 2015), periportal hepatocytes expressing Sox9 or Mfsd2a (Font-Burgada et al. 2015; Pu et al. 2016), distributed Tert+ hepatocytes (Lin et al. 2018), or broadly distributed hepatocytes with **predominant proliferation of midlobular hepatocytes** (Chen et al. 2020), and the highly proliferative hepatocytes in the midlobular region (He et al. 2021; Wei et al. 2021).

**Hepatocyte proliferation**

A recent study from Willenbring’s group **based on random lineage tracing showed that in liver homeostasis proliferating hepatocytes can be found in all zones but are enriched in the midlobular zone** (Chen et al. 2020). Chen et al. used AAV8-TBG-Cre virus and Rosa26-Rainbow mice to sparsely label hepatocytes. After 13 months of tracing in homeostatic liver, 90% of labeled cells remained single cells, 9% of clones contained 2 cells, and 1% of clones consisted of > 2 cells. **Clones >2 cells were mostly located in the midlobular zone; pericentral and periportal clones almost exclusively consisted of 1 or 2 cells** (Chen et al., 2020).

The below 2 sentences need to be removed.

**Hepatocyte proliferation**

“The above lineage tracing studies focused only on a subset of hepatocytes and their expansion/proliferation, which lacks direct comparisons of different zonal hepatocytes expansion or proliferation in the liver lobules.”

“Until recent, two groups, examining the proliferation of hepatocytes in all zones, reported that midlobular zone hepatocytes contribute to hepatocytes renewal during liver homeostasis (He et al. 2021; Wei et al. 2021). “

The original article (Pu and Zhou 2022) has been updated.
